# Probiotics: A multifaceted approach to health promotion-from disease prevention to food enrichment and delivery systems

**DOI:** 10.3934/microbiol.2025026

**Published:** 2025-07-22

**Authors:** Srirengaraj Vijayaram, Vivekanandan K.E, Sabariswaran Kandasamy, Hary Razafindralambo, Einar Ringø, Yun-Zhang Sun, Gayathri Kaliyannan

**Affiliations:** 1 Xiamen Key Laboratory for Feed Quality Testing and Safety Evaluation, Fisheries College, Jimei University, Xiamen 361021, China; 2 Centre for Global Health Research, Saveetha Medical College and Hospital, Saveetha Institute of Medical and Technical Sciences, Saveetha University, Thandalam, Chennai- 602 105, Tamil Nadu, India; 3 Department of Microbiology, PSG College of Arts and Science, Coimbatore- 641014, Tamil Nadu, India; 4 Department of Biotechnology, PSGR Krishnammal College for women, Avinashi road, Peelamedu, Coimbatore-641004, Tamilnadu, India; 5 ProBioLab, Campus Universitaire de la Faculté de Gembloux Agro-Bio Tech/Université de Liège, B-5030 Gembloux, Belgium; 6 BioEcoAgro Joint Research Unit, TERRA Teaching and Research Centre Microbial Processes and Interactions, Gembloux Agro-Bio Tech/Université de Liège, B-5030 Gembloux, Belgium; 7 Norwegian College of Fishery Science, Faculty of Bioscience, Fisheries, and Economics, UiT the Arctic University of Norway, Tromsø, 9037, Norway; 8 Department of Biomaterials, Saveetha Dental College and Hospital, Saveetha Institute of Medical and Technical Sciences (SIMATS), Saveetha University, Chennai 600 077, India

**Keywords:** Clinical applications, encapsulation, food supplements, immune system, gut microbiota, probiotics

## Abstract

Probiotics are living microbes that impart overall health benefits when introduced appropriately. They play important roles in activating the immune system, inhibiting pathogens, balancing gut microbiota, providing relief from inflammatory diseases, and helping prevent chronic conditions such as cancer. In this manuscript, we address the multifaceted uses of probiotics in medicinal, food, and cosmetic industries, emphasizing new encapsulation techniques that improve their stability and effectiveness. Demonstrating their uses in food enrichment, disease prevention, and delivery systems, the manuscript offers valuable recommendations for the use of probiotics in different fields. It also anticipates future directions, such as the invention of new encapsulation techniques, the use of probiotics as personalized nutrition, and the application of their therapeutic benefits to new areas such as metabolic and neurodegenerative diseases. The paper demonstrates the potential of probiotics as promising candidates for the promotion of animal and human health in the modern era.

## 1. Introduction

Probiotics have become a crucial component in the development of functional foods and are well-known for their beneficial health effects. [1],[2]. Lilly and Stillwell [3] were the pioneers in defining probiotics as living microbes that provide health advantages to the host. The Food and Agriculture Organization (FAO) and the World Health Organization (WHO) subsequently defined probiotics as "living microorganisms that, when administered in adequate amounts, offer health benefits to the host" [4]. The word "probiotic," coming from the Latin pro and the Greek βιο (meaning "for life"), was first used by Werner Kollath in 1953. He described probiotics as biologically active substances that play a crucial role in promoting health [5]. Various bacterial genera have been recognized as possible probiotics, including *Pediococcus*, *Lactococcus, Enterococcus, Streptococcus, Propionibacterium*, and *Bacillus* [6]. Nonetheless, the predominantly utilized strains are from *Lactobacillus* and *Bifidobacterium* [7], which are involved in the biotransformation of mycotoxins present in food [2], the production of essential vitamins such as vitamin K, riboflavin, and folate [2],[7], and the fermentation of non-digested dietary fibers in the colon [8]. Probiotics additionally promote host health by preventing the establishment of harmful microorganisms. This is achieved via competitive inhibition at mucosal binding sites and the adjustment of host immune responses, thereby enhancing intestinal barrier function [9]. Probiotics are especially beneficial in managing clinical issues like irritable bowel syndrome, inflammatory bowel disease, obesity, Parkinson's disease, diabetes, Alzheimer's, colic in infants, and rheumatoid arthritis [10]–[17]. Probiotics offer health benefits such as the regulation of gut microbiota, reduction of lactose intolerance symptoms, improved absorption of macro and micronutrients, and a lower incidence of allergic reactions in sensitive individuals [18]. Probiotics are usually obtained from fermented foods or dietary supplements, including dairy and non-dairy options [19]. They likewise aid in preserving a healthy gut microbiome and have clinical uses, such as treating or preventing issues like *Helicobacter pylori* infection, diarrhea, hypertension, ventilator-associated pneumonia, acute pancreatitis, diabetes, migraines, autism, and colon cancer [20]. Probiotics can greatly affect the makeup of specific gut microbes, although they may not change the whole microbial community. They have antimicrobial characteristics associated with their generation of hydrogen peroxide, organic acids, ethanol, or protein mechanisms (bacteriocins) [21],[22]. Consequently, probiotics are being incorporated more frequently into various food products, including drinks, yogurt, ice cream, and baked goods. Despite their various advantages, a key difficulty in utilizing probiotics is their sensitivity to food processing conditions and gastrointestinal (GI) stresses. Recent advancements in technology, such as nanoencapsulation and genetic engineering, have greatly enhanced the resilience of probiotic strains in adverse environmental and physiological conditions [23]. They have antimicrobial characteristics associated with their generation of hydrogen peroxide, organic acids, ethanol, or protein-based mechanisms (bacteriocins) [24]. The advancement of microencapsulation methods is essential for safeguarding probiotics against environmental stressors [22]–[26]. Probiotic therapy is essential for promoting healthy growth in both humans and animals. In recent decades, probiotics and other beneficial microorganisms have significantly enhanced human health and the quality of food and nutrition. Although the health benefits of probiotics vary depending on the strain, food products should contain approximately 10^6^ live bacteria per gram or milliliter [27]. Probiotics have demonstrated promising effects in the prevention and management of various clinical disorders, including rheumatoid arthritis, obesity, Parkinson's disease, diabetes, Alzheimer's disease, infantile colic, and irritable bowel syndrome [11]–[17],[28]. The applications of probiotics extend beyond human health, playing a key role in livestock, aquaculture, and poultry management, as well as in the prevention and treatment of both communicable and noncommunicable diseases, such as bacterial, viral, parasitic, or fungal infections, nervous system disorders, obesity, cancer, and allergic conditions. They also show promise in pre-operative and post-operative processes. Recently, probiotic-rich diets have become vital, with elevated levels of probiotic consumption through animal products, fermented fruits, juices, and other food items [29]. Numerous studies have highlighted the role of probiotics in preventing and managing inflammatory diseases and allergic disorders such as atopic dermatitis and rhinitis, as well as in the prevention of diarrheal diseases, infection control, and as natural antibiotics. Probiotics have also been shown to aid in the treatment or prevention of colon and bladder cancers [30],[31]. In this review, we emphasize the gaps, such as the multifaceted role of probiotics in health promotion, focusing on their mechanisms in disease prevention, potential to enhance food products, and the emergence of innovative delivery systems. We mostly contribute comprehensive insights into probiotics, including their types, methods of administration, encapsulation in functional foods, and their clinical applications for the treatment or prevention of various diseases, and provide a comprehensive analysis of the mechanisms involved and the current perspectives on the therapeutic applications of probiotics. We also delve into their safe integration into major food matrices and assess their role in preventing diseases and promoting health.

## 2. Characteristic features of probiotic bacteria

The identification and functional attributes of probiotics are important criteria for the identification of beneficial microorganisms that enhance human health. Probiotics are consumable, non-toxic, and non-pathogenic. Significant features are their acid and bile salt resistance, which enables them to survive the harsh environment of the digestive tract [32]. In addition, probiotics must have the ability to survive and live on both the gastrointestinal and urinary tracts and find niches in them to exert favorable health benefits. Probiotics must be stable and remain alive for long storage, particularly in fermentation and food manufacturing. Another important feature of these microbes is their ability to synthesize antimicrobial compounds that help to repress pathogenic microorganisms [33]. They regulate the host immune system, promoting a balanced immune reaction and enhanced health [34]. In addition, probiotics play a role in repairing or replacing gut microflora, assisting in the maintenance of a healthy intestinal environment. Coupled with their microbial benefits, probiotics have been found to reduce cholesterol, providing cardiovascular protection [35]. They exhibit anti-carcinogenic and anti-mutagenic activities, which help in inhibiting cancer [36]. Probiotics are also known to reduce symptoms of irritable bowel syndrome (IBS) and yield therapeutic relief to individuals with gastrointestinal discomfort. Probiotics play a critical role in inhibiting the development of pathogenic organisms and hence promoting a healthy microbial balance and protecting the host from infections.

## 3. Probiotics: Possible mode of action

The mechanisms through which probiotics operate involve several important processes that benefit the host. These include the release of antimicrobial substances, regulation of the immune system, reduction of allergic infection risks, enhancement of intestinal barrier functions, modulation of gene expression, suppression of harmful organisms, and the production of functional proteins like lactase and natural enzymes [31]. Probiotic microorganisms are decisive for improving the microbial stability within the host and protecting the intestinal epithelial membranes from detrimental pathogens. They produce short-chain fatty acids (SCFAs) such as lactic acid, propionic acid, butyric acid, and acetic acid, which help them thrive in low pH environments, regulate the host's immune response, and inhibit pathogen growth, highlighting the essential qualities of probiotics. These microorganisms generate antimicrobial substances that combat infections, prevent pathogen adhesion, address nutrient deficiencies, eliminate toxin receptors, and modulate the host's immune system. The action mechanisms of probiotics can be summarized as follows: Preventing pathogenic microorganisms from adhering to the intestinal epithelium, regulating SCFA production, enhancing immune responses, modulating lipid metabolism, and suppressing intestinal pro-inflammatory cytokine activity [37]–[39]. The effects of probiotics are closely linked to the species of organisms present in the gut. They promote host health by managing gut mucosal immunity, inhibiting pathogenic microorganisms, improving the gut microenvironment, strengthening the intestinal barrier, reducing inflammation, and enhancing antigen-specific immune responses [40],[41]. Maintaining the intestinal barrier is crucial for ensuring intestinal integrity and function. GIT microbiota composition varies between individuals, with beneficial or pathogenic microbes engaging in symbiotic relationships. Probiotics can promote an increase in beneficial gut microbiota and inhibit pathogenic or opportunistic microbes. Moreover, probiotic therapy is an effective modern approach to treating intestinal infections, exerting its action through various mechanisms, including the production of antimicrobial substances, competition for nutrient substrates, competitive exclusion, enhancement of intestinal barrier function, and modulation of the immune system [42]. Probiotics bring about host health through several mechanisms, including intestinal microbiota modulation, immune homeostasis, enhancement of epithelial barrier function, and gene expression. The release of antimicrobial compounds (e.g., bacteriocins, organic acids, reuterin) that suppress pathogens like *Staphylococcus aureus*, *E. coli*, *Salmonella*, and *Clostridioides difficile* by disrupting membranes and inhibiting adhesion is one of the major mechanisms. They also bring innate immunity by stimulating Paneth cells to release antimicrobial peptides like defensins and lysozyme [43] (Table 1).

**Table 1.** Probiotics and their influence on plants and animals, and their key benefits.

Type	Microorganisms	Examples	Key Benefits	References
Lactic Acid Bacteria (LAB)	*Lactobacillus*	*L. acidophilus, L. casei, L. rhamnosus, L. plantarum*	Improves digestion, enhances immunity, and produces lactic acid	[44]–[46]
	*Bifidobacterium*	*B. bifidum, B. longum, B. breve*	Colon health modulates the immune system and battles gastrointestinal infections	[47],[48]
	*Streptococcus*	*S.thermophilus*	Supports lactose digestion and is used in dairy fermentation	[49]
	*Enterococcus*	*E. faecium, E. durans*	Gut health (especially in animals) inhibits pathogens	[50]
Spore-Forming Bacteria	*Bacillus*	*B. subtilis, B. coagulans, B. clausii*	Heat-stable, supports gut flora, and is used in food and animal probiotics	[51]
Yeast Probiotics	*Saccharomyces* (Yeast)	*S. boulardii*	Prevents antibiotic-associated and traveler's diarrhea, and restores microbiota	[52]
Next-Generation Probiotics	*Akkermansia*	*A. muciniphila*	Supports metabolic health and may reduce obesity and insulin resistance	[53]
	*Faecalibacterium*	*F. prausnitzii*	Anti-inflammatory, associated with a healthy gut in IBD (Inflammatory Bowel Disease) patients	[54]
	*Clostridium*	*C. butyricum*	Produces butyrate and supports mucosal barrier integrity	[55]
Synbiotics (Probiotics + Prebiotics)	Various	*L. rhamnosus + inulin*, *B. longum + FOS*	Enhances probiotic survival and colonization and provides synergistic gut health benefits	[56]

Probiotics stimulate host pattern recognition receptors (e.g., TLRs), modulating NF-κB and MAPK signaling to suppress proinflammatory cytokines (e.g., TNF-α, IL-6) and induce anti-inflammatory mediators like IL-10 and TGF-β. Probiotics stimulate Tregs and modulate the Th1/Th2/Th17 axis, promoting immune tolerance and mucosal immunity (e.g., increased sIgA, NK cell activation). Probiotics in allergy prevention restore microbial stimuli critical for immune maturation, suppress IgE responses, and promote Treg differentiation, improving eczema and allergic rhinitis [57].

**Figure 1.**
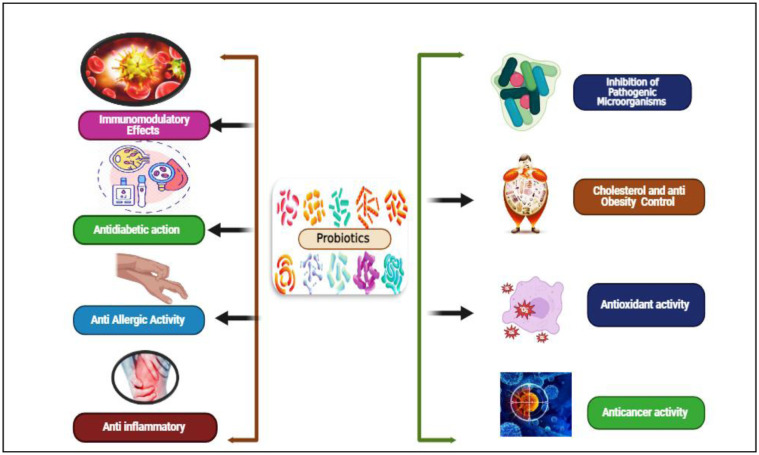
Probiotics application in the prevention and control of diseases.

Probiotics at the gut barrier increase tight junction proteins (e.g., occludin, claudins) and mucins (e.g., MUC_2_), reducing intestinal permeability. Competitive exclusion also prevents colonization by pathogens by occupying the binding sites and limiting the accessibility of nutrients. Molecularly, they modulate gene expression, downregulate inflammatory genes, and enhance epithelial survival through probiotic-derived proteins (e.g., p40, p75) and surface layer proteins. Some strains like *Lactobacillus* and *Bifidobacterium* inhibit pathogen invasion and toxin action, maintain gut homeostasis, and reduce antibiotic-associated effects. Growing evidence is connecting probiotics with systemic actions via microbiota–gut–organ axes, impacting respiratory and neurological well-being, with possible therapeutic applications in treating infections such as COVID-19 [58] (Table 2 and Figure 1).

**Table 2.** Applications of probiotics.

Probiotic strain	Dose and administration	Study type/model	Response	Application	References
*Bifidobacterium lactis* Bb-12 and *Lactobacillus rhamnosus* GG (LGG)	Oral administration of 3 × 10^8^ CFU of LGG and 10^9^ CFU of *B. lactis* for 4 weeks	RDBS with 27 infants with atopic disease	After 2 months, a major improvement in the skin condition occurred in the probiotic group. (SCORAD) and the attentiveness of soluble CD4+ decreased in the probiotic groups	Atopic eczema	[59]
*Lactobacillus fermentum* VRI-033 PCC	1× 10^9^ CFU twice daily for 8 weeks	RDBPS with 56 children	A decrease in the SCORAD indicator was seen in the probiotic-treated group. After the study, more children treated with this probiotic had milder atopic dermatitis	Atopic dermatitis	[60]
*Lactobacillus* F19	1 ×10^8^ CFU/day for 4–6 months	RDBPS with 179 infants	The increasing occurrence of eczema at 13 months was lower in the probiotic group. At 13 months, the INF-y/IL-4 percentage was higher in the probiotic group. No differences in serum concentrations of IgE	Eczema	[61]
*B. infantis* BB-02, *Streptococcus thermophilus* TH-4, and *B. lactis BB*-12	A mixture of *B. infantis* BB-02 (300 ×10^6^ CFU), *S. thermophilus* TH-4 (350 ×10^6^ CFU), and *B. lactis* BB-12 (350 × 10^6^ CFU). Total: 1 × 10^9^ CFU per 1.5 g in a powder once daily, until discharged from the hospital or term-corrected age	MDPR with 1099 very preterm infants	There were no differences in the occurrence of eczema between the two groups. Similarly, the incidences of atopic eczema, food allergy, wheezing, and atopic sensitization were comparable in both groups.	Eczema, atopic sensitization, food allergy, and wheezing	[62]
Mixture of *L. casei, L. rhamnosus, L. plantarum*, and *B. lactis*	Oral administration at 2 × 10^9^ CFU in each strain, twice daily for 6 weeks	DBPS with 100 children	The combination of probiotics did not inhibit the growth of other strains, but no changes in clinical development were seen between the treated and placebo groups	Atopic dermatitis	[63]
*Lactobacillus pentosus* B281 *Lactobacillus plantarum* B282	NM	NM	Cell proliferation and Cell cycle arrest (G1)	Caco-2 and HT-29	[64]
*Bacillus polyfermenticus* KU3	NM	NM	>90% ↓ Cell proliferation	LoVo, HT-29, AGS	[65]
*Lactobacillus plantarum* A7 *Lactobacillus rhamnosus* GG	NM	NM	↓ Cell proliferation	Caco-2, HT-29	[66]
*Lactobacillus paracasei* IMPC2.1 *Lactobacillus rhamnosus* GG	NM	NM	↓Cell proliferation and Induction of apoptosis	DLD-1, HGC-27	[67]
*Bacillus polyfermenticus*	NM	NM	↓Cell colony formation in cancer cells (N/E on normal colonocytes)	NMC460	[68]
*Lactobacillus plantarum* species 299	Colitis was induced by 30 mg (0.6 mL of 5% aqueous solution) of TNBS. 7 days supplementation of 10^9^ colony forming units (CFU) of *Lactobacillus plantarum* (in oat fiber) following colitis initiation.	Rat model (TBSN)	No positive effects on the rat's gut permeability, weight changes, colon microscopic scores, and the level of blood albumins;	CD-like	[69]
*Bifidobacterium bifidum* PRL2010	daily oral supplementation of 10^9^ of *B. bifidum* PRL 2010. Colitis (TNBS 2.5 mg/mice) was induced in the 5th day of probiotic strain feeding.	Murine model (TNBS)	Reduction in the edema, decrease in the macroscopic damage and histological scores, decrease in weight loss, and anti-inflammatory effects	CD-like	[70]
*Bifidobacterium animalis* subsp. lactis BB12	7 days supplementation (twice a day) with 1.2 × 10^10^ CFU *Bifidobacterium animalis* subsp. lactis BB12 by oral gavage before colitis. Colitis was induced by 3% DSS added to drinking water for 6 days.	Murine model (DSS)	Defense against a reduction in colon length, better picture of the colon histology, decrease in apoptosis in the epithelial layer, reduction in the level of TNF-ά;	UC-like	[71]
*Lactobacillus delbrueckii*	Colitis was induced by administering 3% (w/v) dextran sulfate sodium (DSS) in the drinking water for 7 days. Probiotic treatment (5x10^9^ ) CFU in mice began one day before colitis induction and continued until sacrifice.	Murine model (DSS)	Regulation of the NF-kB pathway and a decrease in the inflammatory state	UC-like	[72]
*Saccharomyces boulardii*	Patients with CD in remission (based on Crohn's disease activity index) were supplemented with *S. boulardii* about 4 ×10^8^ cells every 8 h as an oral capsule formulation during 3months.	Human model	Useful to regulate remission and bowel sealing	CD	[73]
*L. rhamnosus* GG, *B. lactis*	(10^10^CFU) 1 capsule per day	RDPBS	↓WC (p < 0.001)	obesity and overweight	[74]
*L. salivarius* UCC118	(10^9^ CFU) 1 capsule per day	RDPBS	No effect	obesity and overweight	[75]
*L. rhamnosus* GG	(10^10^ CFU) 1 capsule per day	RDPBS	Probiotic administration may decrease weight gain, particularly up to 4 years of age (p = 0.08)	obesity and overweight	[76]
*L. acidophilus, L. rhamnosus, B. bifidum, B. longum* and *E. faecium*	(each 4.3 ×10^8^ CFU) and (8.2 × 10^8^ CFU)	Open-label, randomized, controlled trial	↓BMI (p < 0.05), ↓ HC, and WC (p < 0.05)	obesity and overweight	[77]
*L. casei, L. rhamnosus, S. thermophilus, B. breve,L. acidophilus, B. longum* and *L. bulgaricus*	(Each 2 ×10^8^ CFU), vit. E, A, C per day	RTBS	↓BMI, ↓ WC	obesity and overweight	[78]
*Lactobacillus acidophilus* NCFM	NM	NM	Reduces the risk associated with type 2 diabetes mellitus and increases host's metabolic system, ensuring weight administration	Diabetes and obesity	[79],[80]
*Lactobacillus gasseri* SBT2055	NM	NM	Majorly reduces in body mass index (BMI), waist, abdominal Visceral Fat Area (VFA), and hip circumference.	Diabetes and obesity	[81]
*Enterococcus faecium, Streptococcus thermophilus*	NM	NM	Reduces body weight, systolic Blood Pressure LDL-C (Low-Density Lipoprotein Cholesterol), and increases fibrinogen levels.	Diabetes and obesity	[82]
*Bifidobacterium animalis* DSMZ 23733, *Bifidobacterium breve* DSMZ 23732	NM	NM	Decreases total cholesterol level	Diabetes and obesity	[83]
*Lactobacillus* species	NM	NM	Noticeably increases inhibitory antagonistic effect, and a main target for pathogens (Gram-positives and Gram-negatives) and food spoilage microorganisms	Antagonistic activity	[84],[85]
*Lactococcus lactis* subsp Cremoris and Lb. brevis	NM	NM	Two dissimilar, primarily linear peptides, with or without post-translational modifications at the C-terminus, were attached as required.	Antagonistic activity that is	[86],[87]
*Lactobacillus kefir, Lactobacillus kefiranofaciens*, and *Lactobacillus kefirgranum*	NM	NM	The production of bacteriocin increases the antibacterial activity of intestinal epithelial cells. They also reduce inflammation and serum cholesterol levels and produce an EPS known as kefiran.	Antibacterial,anti-cholesterol, and anti-inflammation	[88]–[90]
*Lactobacillus plantaFrum*	NM	NM	Antioxidant activity	Antioxidant activity	[91]–[93]
*Lactobacillus plantarum, Lactobacillus fermentum*, and *Saccharomyces cerevisiae*	NM	NM	Antibacterial activity against pathogens	Antimicrobial activity	
*L. fermentum* CECT5716	NM	NM	Increases antibody production against the Influenza virus	Antiviral activity	[94]
*L. plantarum* YML009	NM	NM	triggering of Th1 immune response against H1N1 Influenza virus	Antiviral activity	[95]
*L. rhamnosus* GG	NM	NM	Production of IFN- and Ils against Respiratory syncytial virus (RSV)	Antiviral activity	[96]
*E. faecium* NCIMB 10415	NM	NM	Promotion of nitric oxide (NO) production and secretion of Interleukins (IL-6 and IL-8) against Transmissible gastroenteritis virus) TGEV	Antiviral activity	[97]
*Lactobacillus* sp.	NM	NM	Rule of blood pressure	Immune system	[98]
*Bacillus licheniformis*	NM	NM	Decrease the effect of antibiotics use in the treatment of diarrhea and can detoxify aflatoxin B1 up to 94.7% in food matrices.	Antibiotic	[99]
*Bifidobacterium longum* CMCC P0001	NM	NM	Action of gastrointestinal disorders	Antibiotic	[100]
*Lactobacillus*, *Bifidobacterium*	NM	NM	positive effects on mental health and mood.	Depression, Anxiety, and Mental Disorders	[101]
*Lactobacillus acidophilus* ATCC4495, *Lactobacillus plantarum* NRRL B-4496	NM	NM	Important antifungal activity	Antifungal activity	[102]
*Lactobacillus buchneri*	NM	NM	Antagonistic activity against *Candida albicans*	Antifungal activity	[103]
*Weissella cibaria* and *Weissella koreensis*	NM	NM	Antimicrobial activity against *L. monocytogenes*, *E. coli*, and *Salmonella spp*	Anti-microbial activity	[104]
*Lactobacillus acidophilus, Lactobacillus delberukii*	NM	Gnotobiotic mice	Digest lactose and sugar in milk and other dairy products	Lactose intolerance	[105]
*Lactobacillus reutri, Lactobacillus acidophilus, Lactobacillus rhamnosus GR-1*	NM	Murine	Stop the colonization of pathogenic bacteria	Urinary tract infection	[99],[106],[107]
*L. casei* DN 114001	NM	Human	Cost-efficient way to treat antibiotic-associated diarrhea during antibiotic treatment	Antibiotic treatment	[108]
*L. acidophilus B. animalis subsp. lactis L. delbrueckii subsp. bulgaricus S. thermophilus*	NM	Clinical trials	Clinical trials on adults (avg. age, 39) who were tested for 12 days in three dissimilar groups: bio yogurt (n = 131), commercial yogurt (N = 118), and no yogurt (N = 120). The percentages of participants suffering AAD during this study are 6.9% (bio yogurt), 11.0% (commercial yogurt), and 14.2% (no yogurt), respectively.	Antibiotic associated diarrhea (AAD)	[109]
*L. casei* ATCC 393	NM	Murine (CT26) and human (HT29) colon carcinoma cell lines	Positive effect: Tumor inhibitory, anti-proliferative, and pro-apoptotic response	Anti-cancer	[110]
*L. gasseri* SBT2055	NM	Human	Boost of fat emulsion droplet size and the repression of lipase-mediated fat hydrolysis	Anti-cholesterol	[111]
*L. helveticus* NS8	NM	Caco-2 Cells and BALB/c mice	Probiotics have immunomodulatory properties	Immune system	[112]
*L. rhamnosus* HN001 and *L. acidophilus* GLA-14	NM	Human	Antimicrobial performance against pathogens responsible for both bacterial vaginosis and aerobic vaginitis	Antimicrobial activities	[113]
*L. plantarum* CRL 725	NM	NM	Riboflavin production in foods	Food	[114]
*L. plantarum S48 and P1201*	NM	NM	Conjugated linoleic acid production in foods	Food	[115]
*B. animalis IM386 + L. plantarum MP2026*	NM	NM	Lactose intolerance	Food	[116]
*Saccharomyces boulardii*	NM	Human	Avoidance of antibiotic-associated diarrhea; β-lactamins or tetracyclins and Miscellaneous, Gastroenteritis	Antibiotic	[117]
*Akkermansia muciniphila*	NM	NM	Anticipation of obesity and metabolic disorders	Obesity and weight loss	[118],[119]

**Abbreviation:** Decrease; human colonic cancer cells: Caco-2, HT-29, SW1116, HCT116, SW480, DLD-1, LoVo; human gastric adenocarcinoma cells: AGS; *Mus musculus* colon carcinoma cells: CT26; HGC-27, HGT-1; human colonic epithelial cells: NMC460; UC- ulcerative colitis; CD-Crohns disease; WC—waist circumference; HC—hip circumferences; RDBS-Randomized, double-blind study; RDBPS-Randomized, double-blind, placebo-controlled study; Scoring Atopic Dermatitis(SCORAD); MDPR-multi-center, double-blind,placebo-controlled, RTBS randomized trial; Randomized triple-blinded controlled trial L–*Lactobacillus*; B–*Bifidobacterium* and *Bacillus*; E–*Enterococcus* and *Escherichia*; W–*Weissella*; S–*Saccharomyces*, *Streptococcus*, and *Salmonella*; C–*Candida*; NM–Not mentioned

## 4. Probiotics action in antimicrobial activity

Numerous studies have indicated that the use of probiotics effectively curtails the growth of pathogenic microorganisms and enhances the immune function of the host [120]–[124]. As research evolves, innovative probiotic strains with specific antimicrobial properties are being recognized, providing promising applications in both clinical and functional food domains. Probiotics exhibit antimicrobial properties through various mechanisms, functioning at several biological levels. These mechanisms include competitive exclusion, where probiotics compete with pathogens for limited receptors on the epithelial surface, as well as for nutrients [125],[126]. Probiotics show broad-spectrum antimicrobial activity through a variety of synergistic mechanisms that operate at molecular, cellular, and ecological levels [127]. A primary mode of action involves the production of antimicrobial substances such as organic acids (e.g., lactic and acetic acids), which decrease the local pH and create an inhospitable environment for pathogenic microbes. Probiotics exert antimicrobial effects through a multifaceted array of mechanisms that function across molecular, cellular, and ecological levels. These beneficial microbes, mostly strains of *Lactobacillus* and *Bifidobacterium*, produce a range of antimicrobial compounds such as bacteriocins, organic acids (like lactic and acetic acid), and hydrogen peroxide, which directly inhibit pathogenic bacteria by disrupting their membranes or metabolic functions [128]. Probiotics also engage in competitive exclusion by occupying epithelial binding sites and outcompeting pathogens for essential nutrients, thereby preventing their colonization. They enhance host immunity by modulating cytokine profiles, promoting anti-inflammatory responses, and strengthening epithelial barrier integrity through the upregulation of tight junction proteins [129]. Furthermore, some probiotics interfere with quorum-sensing pathways of pathogens, disrupting microbial communication and suppressing virulence gene expression. They also contribute to restoring and maintaining a balanced microbiota composition, which indirectly limits the proliferation of opportunistic pathogens. Emerging evidence suggests that certain probiotics possess antiviral and antifungal capabilities, mediated by the secretion of bioactive molecules such as exopolysaccharides. Collectively, these multifactorial mechanisms underscore the potential of probiotics as effective agents for antimicrobial defense and microbiome regulation. They also compete with pathogens for adhesion sites and nutrients in the gut epithelium, a process known as competitive exclusion. Additionally, probiotics enhance the host's mucosal barrier integrity and modulate immune responses, promoting the production of anti-inflammatory cytokines and immunoglobulins that further suppress pathogen colonization. Studies emphasize the importance of strain-specific effects and the need for robust in vitro and in vivo models to evaluate probiotic efficacy under physiologically relevant conditions. They also produce antimicrobial metabolites such as organic acids, bacteriocins, reuterin, and secondary bile acids that can directly suppress pathogen growth [130],[131]. Probiotics can interfere with pathogen virulence mechanisms by preventing biofilm formation and inhibiting the production of toxins and enzymes [132]. Some probiotics, like *Propioniferax innocua*, can break down mature biofilms [130]. Additionally, probiotics enhance the host's innate immunity by stimulating the production of antimicrobial peptides by Paneth cells (They are known for their role in innate immunity and intestinal homeostasis) in the intestine, providing another layer of defense against pathogens [133]. In a nutshell, probiotics employ a multi-faceted approach to antimicrobial activity, encompassing direct antagonism, competitive exclusion, and host-mediated effects. These mechanisms contribute to maintaining microbial balance in the gut and strengthening the intestinal barrier against pathogens. However, it's important to note that not all probiotic strains have similar therapeutic effects, highlighting the need for rigorous clinical trials to elucidate strain-specific benefits. Probiotics have been proven to effectively inhibit pathogenic microorganisms and improve the immune responses of the host [120]–[124]. They synthesize bioactive compounds, including bacteriocins, lactic acid, pyroglutamate, and hydrogen peroxide, which specifically target pathogens and stimulate immune pathways [134]–[140]. These compounds are known to inhibit microbes such as *E. coli*, *Salmonella typhimurium*, *Yersinia enterocolitica*, and *Clostridium difficile* [141]. Specific strains like *Lactobacillus fermentum* TcUESC01 and *L. plantarum* ATCC 8014 show strong antimicrobial properties and survival capabilities in intestinal conditions [142],[143]. *Lactobacillus paracasei*, *L. rhamnosus* GG, and *L. acidophilus* KS400 also demonstrate inhibitory effects against various pathogens, including *Salmonella enterica* and vaginal pathogens [144]–[146]. *Bifidobacterium* and *Lactobacillus* species exhibit antimicrobial and antifungal activities, particularly *B. bifidum* PTCC 1644, which inhibits *Aspergillus parasiticus* and reduces aflatoxin levels [147],[148]. Bacteriocins from *Bifidobacterium* further suppress a wide range of pathogens [149]. Probiotics are effective against *Helicobacter pylori*, enhancing immune response, reducing inflammation, and alleviating symptoms, especially through *Lactobacillus* species like *L. reuteri* [150],[151].

## 5. Probiotics action in the immune system

Probiotics significantly influence the host immune system by modulating innate and adaptive immune responses through diverse mechanisms. They regulate the production of cytokines and immunoglobulins, maintaining a critical balance between pro-inflammatory and anti-inflammatory pathways [152],[153]. Probiotics play a crucial role in modulating both innate and adaptive immunity in humans through various mechanisms. These beneficial microorganisms inhabit the intestines and change the composition of the gut microbiota, which subsequently impacts host immunity [154]. Influencing the gut microbiome, probiotics can improve overall immunity and enhance the quality of life for individuals. Probiotics affect innate immunity by stimulating epithelial innate immune responses, such as increasing the production of epithelial-derived TNF-alpha and restoring the function of the epithelial barrier [155]. They also activate NF-kB, which is vital for innate immune responses. Interestingly, this stimulation of innate immunity by probiotics promotes gut health, contrary to the conventional belief that probiotics primarily have an anti-inflammatory effect [155]. In terms of adaptive immunity, probiotics impact T cell and B cell-mediated responses, which can be either beneficial or harmful to the host depending on the context and site of infection [156]. Furthermore, probiotics help regulate intestinal adaptive immunity by maintaining immune tolerance towards symbionts and preserving the integrity of the intestinal barrier [157]. They also modulate DC/NK interactions, balance T-helper cell responses, and promote the secretion of polymeric IgA [158]. Recent research has uncovered that innate immunity has a memory capacity referred to as “trained immunity” [159]. For instance, *Lactobacillus plantarum* O6CC2 enhances Th1 cytokine responses, including interleukin-12 (IL-12) and interferon-gamma (IFN-γ), during influenza infections by activating natural killer (NK) cells, which are essential for early-stage defense [160]. Similarly, *Lactobacillus paracasei* CNCM-I-1518 modifies pro-inflammatory and anti-inflammatory cytokine profiles in the lungs and alters immune cell counts, thereby reducing viral loads and improving outcomes following influenza infection [160]. In enteric infections caused by *Escherichia coli* and *Salmonella*, probiotics such as *Lactobacillus rhamnosus* enhance the expression of toll-like receptors (TLR2 and TLR9) and NOD-like receptors (NLRs), while modulating cytokine expression, including IL-4, IL-6, IL-12, and IL-10, which play pivotal roles in mucosal immunity [161],[162]. Probiotics also regulate cell signaling pathways, such as NF-κB and MAPK, which are vital in controlling immune responses during clostridial infections [163],[164]. Moreover, certain probiotics, such as *Bifidobacterium breve* MCC1274, stimulate IL-8 and IFN production while activating interferon regulatory factor-3 (IRF3), providing protection against viral pathogens [165]. Probiotics are considered potential candidates for stimulating trained immunity, although the exact mechanisms are not fully understood. This concept opens new therapeutic avenues for enhancing innate immune responses through probiotic administration. Probiotics exhibit diverse effects on both innate and adaptive immunity. They stimulate innate immune responses, regulate adaptive immunity, and potentially enhance trained immunity

## 6. Probiotics action in diabetes

Diabetes mellitus (DM), a genetic and environmental metabolic disorder of long duration, is a serious global health concern [166]. It causes cardiovascular system, kidney, eye, and nerve impairment, with an increased risk of heart disease and stroke [167]. Probiotics have been shown to manage type 2 diabetes mellitus (T2DM). Probiotic yogurts containing *Lactobacillus acidophilus* La5 and *Bifidobacterium lactis* Bb12 increase fasting glucose and antioxidant activity [168]. Clinical trials have reported reductions in fasting plasma glucose (by 15.92 mg/dL) and HbA1c (by 0.53%) following probiotic use [169]. A six-week study using a probiotic capsule with *L. acidophilus*, *L. casei*, *B. bifidum*, and *L. fermentum* showed improved insulin sensitivity, gene expression, and lipid profiles [170]. Probiotic use also enhances fasting glucose levels, reduces inflammation, and increases antioxidant capacity. Animal studies further support probiotics in lowering glucose levels, improving insulin resistance, and modulating gut microbiota [171],[172]. In pediatric cases, probiotics help manage obesity and diabetes by regulating gut flora [173]. They also suppress inflammatory cytokines such as TNF-α, IL-1β, and IL-6. In mouse models, probiotic cocktails reduced blood glucose, pancreatic inflammation, and TLR4 expression while modulating cytokines and improving the Bcl-2/Bax ratio [167]. Probiotics have indicated reassuring effects in diabetes management through various mechanisms: they can improve insulin sensitivity and diminish autoimmune responses by modifying the intestinal microbiota, reducing inflammatory reactions, and alleviating oxidative stress. They affect the host by regulating intestinal permeability, the mucosal immune response, and the gut endocannabinoid system related to inflammation and diabetes [174]. Furthermore, probiotics impact energy extraction from food and biochemically alter molecules derived from the host or gut microbes [174]. The anti-diabetic effects of probiotics include the reduction of pro-inflammatory cytokines via the NF-κB pathway, decreased intestinal permeability, and reduced oxidative stress [175]. Short-chain fatty acids (SCFAs) produced by probiotics are crucial for glucose homeostasis by activating G-protein-coupled receptors on L-cells, which encourages the release of glucagon-like peptide-1 and peptide YY, leading to increased insulin secretion and appetite suppression [176].

Some research has shown inconsistent results regarding the effectiveness of probiotics in diabetes management. While many studies suggest improvements in fasting blood glucose, insulin sensitivity, and systemic inflammatory and antioxidant status in type 2 diabetic patients [177], others have pointed out limitations in clinical trials [178]. This emphasizes the need for further research to develop solid probiotic-based adjuvant treatment protocols for diabetes. Probiotics hold promise in diabetes management by modulating gut microbiota, reducing inflammation and oxidative stress, enhancing insulin sensitivity, and regulating glucose metabolism. However, more well-structured human clinical studies are necessary to establish optimal dosages, treatment duration, and the long-term impacts of probiotic interventions in diabetes management [179].

Investigations into the effects of probiotics on diabetes have produced promising findings; however, there are notable gaps in our knowledge of this field. Probiotic supplementation has been correlated with improvements in glycemic control, lipid profiles, and inflammatory markers in individuals with type 2 diabetes [180],[181]. However, inconsistencies in results arise due to differences in probiotic formulations, dosages, and treatment durations [182]. This variability highlights the need for standardized protocols and more comprehensive clinical trials to establish evidence-based treatment guidelines [10]. While many researchers have focused on the influence of probiotics on metabolic parameters, there is a lack of research into the specific mechanisms of action. More investigation is needed to clarify how probiotics impact the gut-skin and gut-skin-brain axes, as well as their effects on intestinal microbiota and host metabolism. Safety evaluations and long-term studies are crucial to identify potential adverse effects and to determine the best dosages for probiotic supplementation [183]. Additionally, the lack of regulatory oversight raises concerns about the quality and labeling accuracy of commercial probiotic products, particularly for at-risk populations [184].

## 7. Probiotics action in obesity and cholesterol

Probiotics have shown promising actions in obesity and cholesterol management, but several gaps in the research have to be addressed. The mechanisms through which probiotics work on obesity are yet to be determined [185]. Even though studies have shown their capacity to modulate gut microbiota, affect lipid metabolism, and affect inflammatory processes, larger trials are warranted to describe the pathways precisely [186],[187]. Large-scale, methodologically sound clinical trials are needed to establish the safety and efficacy of probiotic interventions for the treatment of obesity and lipid control [188]. Longitudinal studies must also be performed to determine the long-term consequences of probiotic supplementation. A severe deficiency in the standardization of study design and methodology is to blame for much of the heterogeneity of results and potential biases [189]. Such limitations must be addressed in future research using stricter and more consistent methods. The interaction between probiotics, gut microbiota, and the gut-brain axis about obesity and cognitive processes must be examined further as well [189]. Obesity, driven by modern lifestyles, is a major global health issue, with projections indicating that by 2030, 38% of adults will gain weight and 20% will be obese [190]. Contributing factors include energy imbalance, sedentary behavior, and gut microbiota composition. The intestinal microbiota influences obesity directly through organ interactions and indirectly via metabolites like short-chain fatty acids (SCFAs) [191]. A higher Firmicutes/Bacteroidetes (F/B) ratio is associated with increased body weight [192]. Probiotics help regulate body weight and adipose tissue by modulating physiological functions and hormone secretion, including leptin and adiponectin. Strains such as *Lactobacillus acidophilus*, *L. casei*, and *Bifidobacterium longum* show hypocholesterolemia and anti-obesity effects through thermogenic and lipolytic activity. *Lactobacillus gasseri* BNR17 reduces adipose tissue and regulates leptin, while *L. acidophilus* and *B. longum* demonstrate similar benefits. *Lactobacillus curvatus* HY7601 and *L. plantarum* KY1032 (5 × 10⁹ CFU/day for over two months) reduced fat accumulation, BMI, and inflammatory markers (IL-1β, TNF-α, IL-6, MCP-1) and enhanced fatty acid oxidation in the liver [193]. *L. rhamnosus* GG and *L. sakei* NR28 also decreased the F/B ratio and reduced obesity-related markers in mice [194]. Probiotics from the *Lactobacillus* and *Bacillus* genera lowered body weight, F/B ratio, and hepatic steatosis in high-fat-diet models [195]. Additionally, *Lactobacillus* species in foods like yogurt improve cholesterol profiles [196]. Probiotics reduce cholesterol by deconjugating bile salts, which hinders lipid absorption [197]. *Lactobacillus reuteri* CRL1098 significantly lowers cholesterol, triglycerides, and the HDL-to-LDL ratio without affecting microbial composition [198]. While probiotics exhibit potential in tackling obesity and cholesterol-related challenges, considerable research gaps remain. Future investigations should concentrate on identifying specific strains and mechanisms of action, conducting extensive clinical trials, and standardizing research methodologies to yield more definitive evidence regarding the effectiveness of probiotics in managing obesity and cholesterol levels.

## 8. Probiotics action in allergic infections and antioxidant response

Probiotics have revealed promising effects across a range of health conditions, including allergic infections and antioxidant responses. However, there are notable research gaps in understanding their mechanisms of action and effectiveness. In terms of allergic infections, while probiotics have indicated potential benefits, the outcomes of clinical studies are in the preliminary stages and need further validation [199]. The specific mechanism by which probiotics prevent pathogen-induced membrane damage and modulate the immune system remains largely unknown [200]. Additionally, inconsistencies in study designs, outcome measures, probiotic strains, dosages, and matrices used in clinical trials make it difficult to reach definitive conclusions [201]. Concerning antioxidant responses, there is a significant gap in research regarding the precise role of probiotics in modulating oxidative stress and antioxidant defense systems across fish species [202]. The variations in experimental design and species-specific responses add to the complexity of this field. Interestingly, while probiotics demonstrate therapeutic potential for immune response-related conditions such as allergies and eczema, their effectiveness in treating bronchial asthma has not been established [203]. Furthermore, the influence of probiotics on specific viruses in respiratory tract infections has not been sufficiently studied [201]. Future research should aim to standardize methodologies, explore species-specific responses, and clarify the mechanisms of probiotic action in allergic infections and antioxidant responses. Long-term studies with larger sample sizes, comparing different probiotic strains and dosages, are necessary to establish strong correlations between dietary interventions and the observed effects [201],[202]. Additionally, further research is needed to understand the interactions between probiotics and other factors. Allergic diseases pose a major global health and economic burden [204]. They result from hypersensitivity reactions of the immune system to allergens, activating mast cells and basophils and releasing allergic mediators. Symptoms range from mild (sneezing, rashes) to severe (anaphylaxis) [205],[206]. WHO identifies several allergic conditions, such as asthma, anaphylaxis, rhinitis, eczema, and hives, as well as triggers like food, drugs, and insect stings. Probiotics are being explored for their anti-inflammatory potential in allergy prevention, though the topic remains debated [207]. *Lactobacillus plantarum* L67, for instance, promotes IL-12 and IFN-γ production [208], while probiotics in general restore gut homeostasis and interact with immune cells to reduce allergy symptoms [209]. They also boost mucosal IgA and activate T and B cells [210]. *Bifidobacterium* species have been shown to lower early eczema and atopic dermatitis risks in infants [211], and *Lactobacillus* strains help prevent respiratory allergies by reducing MMP9 expression and inflammation in lung tissue [212]. Meta-analyses support the role of probiotics in preventing allergic conditions in children [213],[214]. Oxidation is essential for energy production but generates reactive oxygen species (ROS), such as O_2_⁻, H_2_O_2_, and ·OH, which may cause cellular damage if uncontrolled [215]. Probiotics exhibit antioxidant properties through enhancing antioxidase activity, producing antioxidant metabolites, and modulating signaling pathways and gut microbiota. *Lacticaseibacillus rhamnosus* has shown strong antioxidant activity in physically stressed individuals [216], and *Lactobacillus paracasei* spp. *paracasei* YBJ01 improved serum SOD and GSH-Px activity in a dose-dependent manner, with increased hepatic and splenic protein expression in mice [217]. Various probiotics also enhance enzymes like glutathione S-transferase, glutathione reductase, GSH peroxidase, SOD, and catalase, offering protection against oxidative and carcinogenic damage [218].

## 9. Probiotics action in inflammatory bowel disease

Probiotic therapy holds great potential as an alternative management option for inflammatory bowel disease (IBD), but the current evidence remains weak [219]. Meta-analyses of randomized clinical trials indicate that probiotics have a significant impact on reducing symptoms like bloating and flatulence in patients with IBD and improve the quality of life in comparison to control groups [220]. IBD is a collection of chronic inflammatory disorders of the gastrointestinal tract (GIT) with features of diarrhea, fever, abdominal pain, ulcerative colitis, chronic disease progression, weight loss, and nutritional deficiencies, including iron deficiency anemia [221],[222]. Probiotic therapy has the potential to be beneficial in the management of ulcerative colitis, but evidence of its efficacy in Crohn's disease is conflicting [223]. Probiotics have been shown to induce intestinal angiogenesis by vascular endothelial growth factor receptor (VEGFR) signaling, which is of importance in modulating acute and chronic inflammation in intestinal mucosal tissue involved in IBD [224],[225]. Probiotics are important in immune system modulation by lowering inflammation through multiple pathways. They control Toll-like receptor (TLR) and G-protein coupled receptor (GPR) pathways and stimulate anti-inflammatory regulatory agents like A20, Bcl-3, and MKP-1, inhibiting lipopolysaccharide (LPS)-stimulated TLR4 activation [226]. Certain pathways targeted by probiotics include the NF-κB, mitogen-activated protein kinase (MAPK), and pattern recognition receptor (PRR) pathways. Probiotics also block LPS binding to the CD14 receptor, inhibiting NF-κB activation and pro-inflammatory cytokine induction [227]. *Lactobacillus delbrueckii* derived from dairy controls the NF-κB pathway, dramatically attenuating inflammation in a DSS-colitis mouse model [228]. Treatment with Bifidobacterium bifidum increases anti-inflammatory cytokines such as IL-10 but decreases pro-inflammatory cytokines such as IL-1 in the colon [229]. Likewise, *Bifidobacterium* strains inhibit pro-inflammatory cytokines such as IL-8 but induce IL-10 production in peripheral blood mononuclear cells from ulcerative colitis patients [230]. Probiotics have been observed to elevate anti-inflammatory cytokines like IL-10 and IL-12 and lower pro-inflammatory cytokines like IL-1β and IL-6 in different infections [231],[232]. This modulation is attributed to NF-κB and interferon-gamma (IFN-γ) suppression, alteration of cyclooxygenase-2 (COX-2), and elevated secretory IgA [233],[234]. The *Lactobacillus casei* strain greatly decreases IL-6 and IFN-γ production in lipopolysaccharide (LPS)-evoked murine chronic IBD models, improving anti-inflammatory responses [235]. *Lactobacillus gasseri* also has shown greater anti-inflammatory effects against breast cancer by lowering TNF-α and promoting IL-10 production [236]. Administration of live and heat-killed *Lactobacillus plantarum* AN1 has been reported to modulate the composition of gut microbiota and induce anti-inflammatory action in murine models of IBD. The mechanisms involved were found to occur through the generation of nitrogen oxide and exclusion of RAW264.7 cells from the toxic action of hydrogen peroxide, as revealed by in vitro experiments [237]. Probiotics also inhibit LPS binding to CD14 receptors, inhibiting total NF-κB activation and pro-inflammatory signaling [238].

## 10. Probiotics action in cancer disease

Probiotics have shown potential in cancer prevention and therapy by improving gut health and enhancing immune function [239], with colorectal cancer (CRC) being a primary focus [240]. In gastrointestinal cancers, they strengthen the intestinal barrier, lower oxidative stress, and inhibit tumor growth [241]. Some studies also suggest a reduced risk of breast cancer with regular probiotic intake [242], and emerging evidence indicates benefits in oral cancer through immune modulation [243]. In CRC patients, probiotics help regulate gut microbiota by increasing beneficial bacteria and reducing harmful species like *Fusobacterium* [244]. Postoperative gastric cancer patients benefit from reduced inflammation and improved immunity through probiotic use [245]. During chemotherapy, probiotics help restore gut flora, increasing beneficial microbes and reducing harmful ones, thereby improving outcomes [246]–[248]. Further research is needed to evaluate the long-term effects of probiotics and to optimize their use in cancer therapy. Proposed mechanisms include carcinogen degradation, short-chain fatty acid (SCFA) production, regulation of cell proliferation and apoptosis, and enhancement of immune signaling. Additionally, heat-killed probiotics, when combined with radiation therapy, have been shown to suppress cancer cell growth [249]. *Lactobacillus fermentum* strains promote colon epithelial health and inhibit cancer cell growth via SCFAs, while *L. acidophilus* and *L. rhamnosus* also show anti-tumor activity [240]. Probiotic lactic acid bacteria modulate fecal enzymes linked to colon cancer [250], and strains like *Bifidobacterium adolescentis* SPM0212 suppress cancer cell lines [251]. *Lactobacillus* strains produce peptidoglycans that inhibit cancer cells [252] and enhance chemotherapy effects [253]. *E. coli* Nissle 1917 boosts anti-tumor immunity in liver cancer [254], and *L. casei*-derived ferrichrome induces apoptosis via the JNK pathway [255]. *Lactococcus lactis* KC24 exhibits anticancer effects on various cell lines [256]. Clinical and preclinical studies confirm probiotic roles in carcinogen degradation, gut microbiota modulation, and enzyme reduction [257]. Probiotics also combat breast cancer by modulating cytokines [258] and enhancing immune responses [259]. Strains such as *L. rhamnosus*, *L. casei*, *L. paracasei*, and *L. plantarum* suppress cancer cell growth by downregulating ErbB-2 and ErbB-3 [260], with clinical trials supporting improved anticancer outcomes [261].

## 11. Encapsulation of probiotics

**Figure 2.**
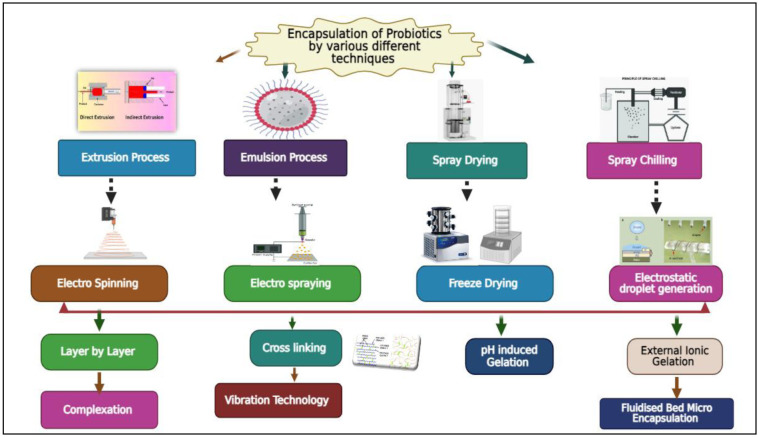
Probiotics are encapsulated in different techniques.

Biopolymers are essential in the microencapsulation process of probiotics for promoting their storage and viability while being transported through the upper gastrointestinal tract [262]. Micro- and nanoencapsulation methods have been efficient in enhancing the viability, stability, and long-term storage of probiotics in food and pharmaceutical production [263]. Several encapsulation techniques, including lyophilization, spray drying, extrusion, coacervation, and emulsion, have been used to attain such results [264]. Probiotic encapsulation, normally conducted using biopolymer beads through extrusion in water-in-oil emulsions, is a strong weapon to preserve probiotic cells under adverse conditions. Methods such as spray drying, spray chilling, spray-freeze drying, freeze drying, extrusion, coacervation, electro-spraying, and fluidized bed encapsulation have found extensive applications to enhance probiotic viability and storage stability [265]. Pectin, for example, is commonly employed to encapsulate probiotics through extrusion and emulsion methods since it promotes gastric and intestinal resistant encapsulation [266]. Novel processes, such as electrospinning and electrospray processes, have been promising to encapsulate probiotics effectively [267]–[269]. The encapsulation processes also prevent degradation of probiotics and their bioactive compounds during food processing and digestion. Encapsulation in bulk solid matrices such as milk protein, lactose, or polysaccharides through techniques, including spray drying, freeze drying, and extrusion, is a standard procedure [270]. Encapsulation is a strong system for encapsulating whole cells or bioactive compounds, such as enzymes, polyphenols, antioxidants, and micronutrients, in capsules for precise delivery. Encapsulation techniques are divided by material size: microencapsulation (3–800 µm) or nanoencapsulation (10–1000 nm). These systems boost bioavailability and safeguard bioactive compounds under suboptimal conditions. For example, xanthan-based encapsulation enhances the viability of probiotics by a significant margin and regulates the targeted release of encapsulated agents [271]. Chitosan-based encapsulation has also been proven to enhance the viability and survival of *Bifidobacterium longum* DD98 in intestinal conditions [271]. Conversely, xanthan–chitosan (XC) blends improve *Bifidobacterium bifidum* survival at temperature conditions of 4 and 25 °C, in contrast to nonencapsulated cells [272]. Alginate-chitosan blends effectively enhance probiotic viability during colon delivery while minimizing porosity [273],[274]. Additionally, new materials like κ-carrageenan have exhibited important advancements in probiotic viability and survival in the gut versus unencapsulated cells [275]. Microencapsulation using κ-carrageenan potentially inhibits *Helicobacter pylori* infection in the stomach [276]. Polysaccharides and protein wall materials are generally utilized for encapsulating probiotics [277]. These substances shield probiotics from poor conditions and boost their resilience, hence minimizing cell loss in hydrocolloid matrices [278],[279]. Various encapsulation materials, such as xanthan, chitosan, and carrageenan, have proved useful in preserving the viability of probiotics [280]. Microencapsulation is an important tool in creating nutraceutical products and new food carriers for probiotics [281],[282]. It is especially vital for increasing probiotic viability in acidic environments in the stomach [283]. Moreover, multilayer encapsulation techniques provide better survivability of probiotic cells [284],[285] (Figure 2).

## 12. Probiotics action in food supplements

**Figure 3.**
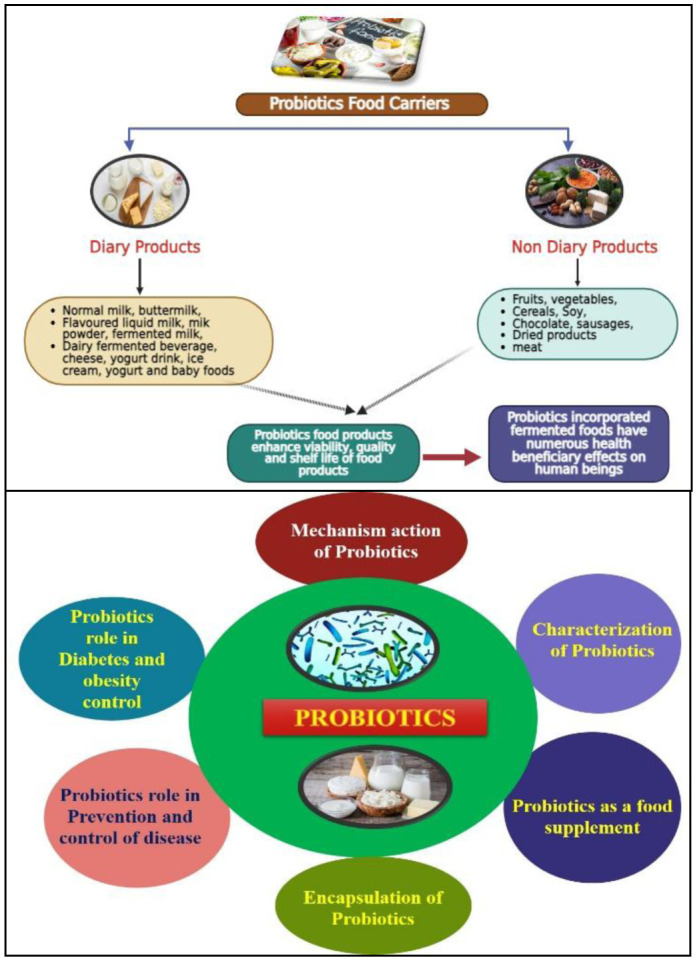
Probiotics supplement in different food products.

Functional foods were initially released by the Japanese government back in 1991, covering a broad spectrum of ingredients like proteins, vitamins, fibers, probiotic bacteria, and other additives to improve human health [286]. Out of these, probiotic food formulations have become a major focus area of study, greatly influencing the future food industry. The market value of probiotic supplements escalated exponentially, from $3.3 billion in 2015 to an estimated $7 billion in 2025. The food and agriculture sectors are changing at a tremendous pace, requiring continuous innovation and technological advancements to enhance the quality of food products [287]. Probiotic functional foods were found to have a beneficial effect on human health [42]. Probiotic bacteria that are ingested in the range of 10⁸ to 10⁹ CFU per gram per day are specifically known to promote physiological functions [288],[289]. Several food ingredients impact the viability and growth of these probiotic bacteria. NaCl and KCl salts, sucrose and lactose sugars, sweeteners acesulfame and aspartame, and artificial coloring and flavoring additives are some of the compounds that can impact probiotic stability. Other factors such as aroma compounds (e.g., diacetyl, acetaldehyde, acetoin), nisin (a polypeptide antibiotic), lysozyme, nitrites, and natamycin also play a significant role in supporting cell viability and growth [290],[291]. Probiotics bring many advantages, such as increased nutritional value, preservation of gut flora, better immune system function, synthesis of antimicrobial substances, and suppression of gut pathogens. Fermented foods with probiotics play a role in these effects by generating peptides, enzymes, antimicrobial compounds, and antioxidants [292]. Meta-analyses and randomized trials show that probiotic supplements are very effective in enhancing human health [293]. Some of the factors that affect the viability of probiotics in food items include conditions of storage, humidity, and temperature. Moreover, probiotics are added to different food items, including yogurt, cheese, milk, cereals, chocolate, sausages, dried products, meat, vegetables, and drinks [294]. Research on probiotics delivered via gastrointestinal-targeted products like drug and food-based formulations is progressing fast. Drug formulations have been more promising than food-based products in some applications [295]. Probiotics improve the shelf life and safety of foodstuffs and are extensively utilized in medical and veterinary applications owing to their safety profile. Fermented foods such as fermented milk products, cheese, and yogurt are popular sources of probiotics [296]. Probiotic strains like *Bifidobacteria*, *Lactobacillus*, and *Streptococcus* are found in fermented food products naturally [297]. Lactic acid bacteria help in food preservation by generating organic acids that prevent the action of spoilage microorganisms. These bacteria are used in several fermented foods, such as yogurt, butter, cheese, kefir, sourdough, brined vegetables, sauerkraut, soy curd, idli batter, uttapam, fermented meat, and drinks [298]–[303]. Maintenance of the growth, viability, and survival of probiotic microorganisms is essential for product formulation, and milk products are the most appropriate carriers in most cases. In recent times, non-dairy products have also become popular for probiotic delivery. For instance, pomegranate juice sustains the growth of *L. acidophilus* and *L. paracasei* [304]. Other non-dairy substrates like fruits, vegetables, cereals, soy, and meat contain necessary nutrients such as proteins, vitamins, dietary fibers, and antioxidants, which qualify them for the development of probiotics [305]. Foods such as milk, buttermilk, flavored liquid milk, milk powder, fermented milk, yogurt drinks, and ice cream continue to be the favored carriers of probiotics. Nevertheless, cereals also demonstrate potential as nutrient substrates, which augment the availability of vitamin B, lysine quality, and diminish non-digestible carbohydrates upon fermentation [306]. Fruit products supplemented with probiotics provide health benefits, a pleasant taste, and added market value [307],[308]. For example, probiotic drinks produced from peach juice fermented using *L. delbrueckii* are ideal for lactose-intolerant consumers [309]. Moreover, olive-derived probiotic beverages are rich in nutrients because they contain organic acids, phenols, antioxidants, and lactic acid bacteria like *L. pentosus*, *L. plantarum*, *L. mesenteroides*, *L. brevis*, and *Pediococcus cerevisiae* [310] (Table 3 and Figure 3).

**Table 3.** Encapsulation of probiotics.

Probiotic strain	Materials	Encapsulation Technology	References
*Saccharomyces boulardii*	Whey	Agglomeration/Spray-drying	[311]
*Lactobacillus delbrueckii*	Soy protein isolate (SPI) and high methoxy pectin (HMP)	Complexation	[312]
*Lactobacillus rhamnosus* GG	Cellulose and chitosan	Crosslinking	[313]
*Lactobacillus plantarum*	Aguamiel, Ag, or sweet whey, SW, as inner aqueous phase	Double emulsion	[314]
*Bifidobacterium animalis* subsp. lactis Bb12	Whey protein concentrate and Pullulan	Electrospinning	[315]
*Bifidobacterium longum* BIOMA 5920	Alginate–human-like collagen	Electrostatic droplet generation	[316]
*Lactobacillus rhamnosus* ATCC 7469	Whey protein isolate/whey protein isolate and inulin/whey protein isolate and inulin and persian gum	Electrospraying/freeze drying/spray drying	[317]
*Lactobacillus plantarum* DKL 109	Na- alginate (Al), alginate/1% gellan gum, Alginate/gum Arabic	External ionic gelation	[318]
*Lactobacillus paracasei* LAFTI® L26, *Lactobacillus acidophilus Ki*, *Bifidobacterium animalis* BB-12, *Lactobacillus casei* -01	Na-alginate	Extrusion	[319]
*Lactobacillus reuteri*	Sweet whey and shellac	Fluidized bed microencapsulation	[320],[321]
*Lactobacillus acidophilus*	Chitosan and carboxymethyl cellulose	Layer by layer	[321]
*Lactobacillus casei*	Sodium caseinate and gellan gum	Ph-induced gelation	[322]
*Bifidobacterium lactis, Lactobacillus acidophilus*	Vegetable fat with lecithin	Spray chilling	[323]
*Lactobacillus reuteri* DSM 17938	Alginates-chitosan	Vibrating technology/extrusion	[324]
*Lactobacillus plantarum*	Whey protein isolate with sodium alginate and denatured whey protein isolate with sodium alginate	Spray drying and freeze drying	[325]
*Lactobacillus plantarum*	k-carrageenan	Emulsification, freeze-drying, or extrusion.	[326]
*Lactobacillus plantarum*	Sodium alginate (SA) PVA	Electrospinning	[327]

## 13. Future perspectives

While traditional probiotic uses in foods and supplements remain relevant, new perspectives are turning probiotics into precision-targeted biotherapeutics. Second-generation probiotics (e.g., *Akkermansia muciniphila*, *Faecalibacterium prausnitzii*) deliver targeted advantage in metabolic, inflammatory, and/or neurodegenerative disease by defined molecular mechanisms. New technologies in personalized nutrition and AI-driven microbiome analysis now enable the tailoring of probiotic regimens to genetic and microbial profiles, and as a result, clinical outcomes are enhanced. Synthetic biology has enabled engineered probiotics to detect disease biomarkers and deliver therapeutic molecules like IL-10 in situ to offer intelligent, targeted treatment. Psychobiotics like *Lactobacillus rhamnosus* act on the gut-brain axis and have the potential to treat mental disease. New delivery technologies like smart encapsulation and stimulus-sensitive systems are improving viability, site-specific action, and neuroprotection.

Aside from the gut, topical probiotics and postbiotics are transforming dermatology by modulating the skin microbiome, but in agriculture and veterinary medicine, they reduce the use of antibiotics and enhance animal and plant resilience. In space medicine, they sustain astronaut microbiota in extreme environments, with engineered strains in progress for long-duration spaceflight. Finally, probiotics are rapidly evolving from general health supplements to complex, microbiome-directed treatments with applications in precision medicine, neurobiology, dermatology, sustainable agriculture, and space health. This is built on mechanistic insight, clinical proof, and new delivery systems that place probiotics at the forefront of next-generation biotherapies.

## 14. Conclusion

Probiotics have become a cornerstone of health promotion, with enormous potential in a wide range of applications in medicine, food, agriculture, aquaculture, and environmental management. This review underscores their roles in disease prevention, immune system modulation, regulation of gut microbiota, and functional food development. The novel encapsulation methods presented here have great potential in promoting the stability and targeted delivery of probiotics and guaranteeing their effectiveness across applications. To tap the full potential of probiotics, researchers should emphasize optimizing encapsulation materials and methods for better viability in production, storage, and delivery. Individualized probiotics customized to unique gut microbiomes are a promising area, with possible applications in treating metabolic disorders, neurodegenerative disorders, and autoimmune diseases. The application of probiotics in sustainable agriculture and aquaculture can also help meet global health and environmental objectives. Probiotic incorporation into daily products and interdisciplinary interactions between microbiology, food science, and biotechnology will be instrumental in further developing their applications. Taking these paths enables probiotics to further develop as indispensable resources for enhancing the health and well-being of human beings, animals, and environments.

Probiotic science is a dynamic and fast-evolving field with enormous potential to modulate gastrointestinal, metabolic, allergic, and infectious diseases. While promising preclinical and early clinical data are being reported, results are largely strain-dependent, context-specific, and derived from small sample sizes and short-duration studies. This again highlights the essential need for high-quality, large-scale clinical trials and standardized protocols to determine efficacy and safety. Mechanistically, probiotics function by competitive exclusion, antimicrobial metabolite secretion, immune modulation, enhancement of the epithelial barrier, and gut-organ axis communication. Next-generation and designed probiotics with precision-targeted action are being developed based on advances in multi-omics and synthetic biology. Postbiotics and paraprobiotics are safer, stable, and immunomodulatory substitutes. However, heterogeneous global regulatory frameworks limit their use in clinical practice. Future cross-disciplinary approaches with clinical research, molecular understanding, personalized microbiome analysis, and regulatory harmonization are needed to reposition probiotics as general supplements to precision biotherapeutics for human health, functional foods, and public health.

## Author contributions

Srirengaraj Vijayaram and Einar Ringø: Conceptualization; writing—original draft; Writing—review and editing, literature search; table preparation. Vivekanandan, Sabariswaran Kandasamy, Gayathri Kaliyannan: Writing, review, and editing. Hary Razafindralambo and Yun-Zhang Sun: Writing, review, and editing; supervision.
